# The power of oral and nasal calls to discriminate individual mothers and offspring in red deer, *Cervus elaphus*

**DOI:** 10.1186/s12983-014-0094-5

**Published:** 2015-01-13

**Authors:** Olga V Sibiryakova, Ilya A Volodin, Vera A Matrosova, Elena V Volodina, Andrés J Garcia, Laureano Gallego, Tomás Landete-Castillejos

**Affiliations:** Department of Vertebrate Zoology, Faculty of Biology, Lomonosov Moscow State University, Vorobievy Gory, 12/1, Moscow, 119991 Russia; Scientific Research Department, Moscow Zoo, B. Gruzinskaya, 1, Moscow, 123242 Russia; Engelhardt Institute of Molecular Biology RAS, Vavilov str., 32, Moscow, 119991 Russia; Animal Science Group. IREC (UCLM-CSIC-JCCM), IDR, Universidad de Castilla-La Mancha, 02071 Albacete, Spain

**Keywords:** Acoustic communication, Individuality, Mother-offspring recognition, Ungulate, Iberian red deer, *Cervus elaphus hispanicus*, Separation calls

## Abstract

**Background:**

In most species, acoustical cues are crucial for mother-offspring recognition. Studies of a few species of ungulates showed that potential for individual recognition may differ between nasal and oral contact calls.

**Results:**

Vocalizations of 28 hinds and 31 calves of farmed Iberian red deer (*Cervus elaphus hispanicus*) were examined with discriminant function analyses (DFA) to determine whether acoustic structure of their oral and nasal contact calls encodes information about the caller’s identity. Contact calls were elicited by brief separation of individually identified animals by a distance over 10 m or by a bar fence. Both oral and nasal calls of both hinds and calves showed high potential to discriminate individuals. In hinds, individuality was significantly higher in the oral than in the nasal calls, whereas in calves, individuality was equally well expressed in both oral and nasal calls. For calves, the maximum fundamental frequency was higher and the duration was longer in oral calls than in nasal calls. For hinds, the maximum fundamental frequency and the duration were indistinguishable between oral and nasal calls. Compared to the pooled sample of oral and nasal calls, separate oral or nasal call samples provided better classifying accuracy to individual in either hinds or calves. Nevertheless, in both hinds and calves, even in the pooled sample of oral and nasal calls, the degree of individual identity was 2–3 times greater than expected by chance. For hinds that provided calls in both years, cross-validation of calls collected in 2012 with discriminant functions created with calls from 2011 showed a strong decrease of classifying accuracy to individual.

**Conclusions:**

These results suggest different potentials of nasal and oral calls to allow the discrimination of individuals among hinds, but not among red deer calves. The high potential of individual recognition even with the pooled sample of oral and nasal calls allows mother and young to remember only one set of acoustic variables for mutual vocal recognition. Poor between-year stability of individual characteristics of hind oral and nasal calls would require updating keys to individual recognition each calving season.

**Electronic supplementary material:**

The online version of this article (doi:10.1186/s12983-014-0094-5) contains supplementary material, which is available to authorized users.

## Background

Mother-offspring recognition is critically important for the survival of the young for many taxa: ungulates [1,2], pinnipedes [3-5], bats [6,7], penguins [8,9], alcids [10,11], larids [12,13] and cranes [14]. Along with the visual and olfactory cues vocalizations play an important role in this process [15-18]. Visual and olfactory cues are generally perceived across a limited area relative to acoustic cues [19], which can also be perceived at night, and through dense vegetation [20]. Vocal recognition is important for selective feeding one’s own offspring [1,3,8,19], protection against predators that are dangerous for the young but not for the mother [21-23], and for maintaining of spatial proximity between mother and young in herds and flocks [24].

Vocal recognition between mother and young is based on individual features of calls. In mammals, the calls are generated by vibrations of vocal folds in the larynx. After that, the sound passes through the vocal tract, which filters the signal, accenting the resonant frequencies of the vocal tract, called formant frequencies [25,26]. So, the vocal output is the joint product of the vocal fold vibrations, determining the call fundamental frequency (f0), and of the work of the vocal tract, determining the values of formant frequencies. According to the source-filter theory, source and filter variables are independent of each other [26,27]. Vocal individual identity can be encoded by call source variables and filter variables. In ungulates, the fundamental frequency has primarily contributed to vocal identity, as exemplified by mother and young fallow deer *Dama dama* [20], red deer *Cervus elaphus* [28], domestic sheep *Ovis aries* [29]; goats *Capra hircus* [17,18], in the young of reindeer *Rangifer tarandus* [30], white-tailed deer *Odocoileus virginianus* [21], mule deer *O. hemionus* [21], and goitred gazelles *Gazella subgutturosa* [31,32]. Formant frequencies play a role in encoding individual identity in the young of goitred gazelles [31] and reindeer [30], in red deer hinds [28], and in mother and young domestic goats [18]. Call duration proved significant in encoding individual identity in mother and young red deer [28] and domestic sheep [29].

Encoding individuality with acoustic variables may be complicated due to variable modes of vocal production. Mother and young ungulates produce two types of contact calls: oral calls that are made through a widely opened mouth, and nasal calls that are made through the nose with a closed mouth. The oral and nasal modes of vocal production were previously reported for the young of white-tailed deer [33] and goitred gazelles [31,34], for mother domestic sheep [35], for mother and young saiga anthelopes *Saiga tatarica* [36], and for mother and young red deer [37]. The acoustic structure of the oral and nasal calls is substantially different [31,35-37]. Oral and nasal calls can be produced by callers in the same series [36,37]. Although it is commonly considered that the oral calls are emitted in situations of higher tension compared to the nasal calls, the communicative potential of these two different call types remains unclear [31,35,36]. In particular, it is unclear whether oral and nasal calls share common cues identifying individuals across types, or hinds and calves must remember two unique sets of cues for encoding individuality specifically in the oral and nasal calls.

Individual vocal traits can be used for recognition only if individual differences remain stable over time. In some species, vocalizations are stable over time [38-40], and mothers can recognize their offspring calls [3,41]. In contrast in others, individual vocal traits change over a short time [42-45]. Although stability of acoustic individuality of offspring calls has been studied [32], to date, no information is available on the stability of mother vocal signature in mammals.

In red deer, individual features were reported for rutting roars of farmed and free-ranging stags [46-48] and for contact calls of mother and offspring [28]. Playbacks showed that calves respond stronger to contact calls of their own mothers compared to alien mothers, whereas hinds respond stronger to contact calls of their offspring compared to calls of alien calves [49]. In a previous study, we described the structure of oral and nasal calls of hinds and calves of Iberian red deer *C.e. hispanicus* during the rut period; however, callers were not individually identified [37].

**Figure 1:**
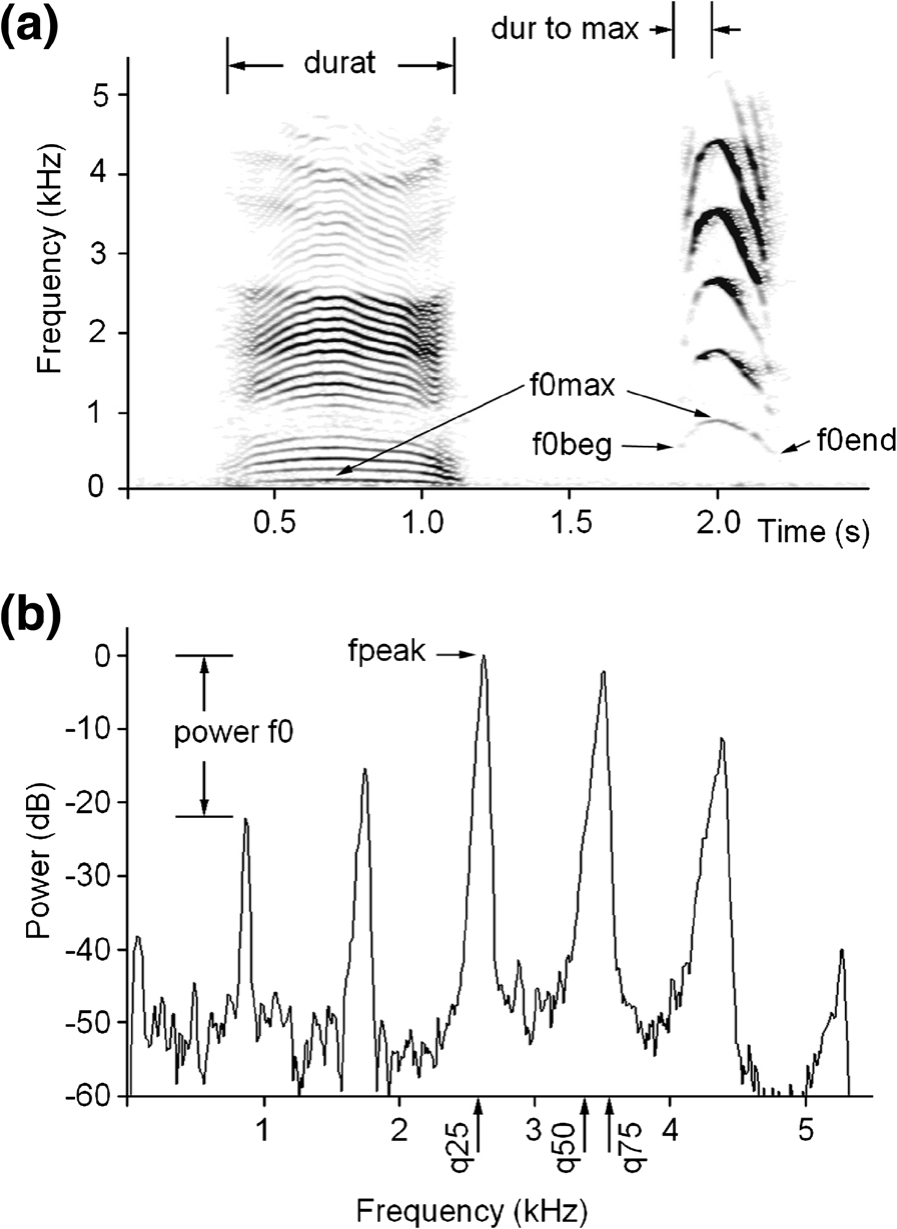
**Measured acoustic variables. (a)** Spectrogram of a hind nasal call (left) and a calf oral call (right). **(b)** Mean power spectrum of 50 ms fragment of a calf call. Designations: durat – call duration; dur-to-max – duration from call onset to the point of the maximum fundamental frequency; f0max – the maximum fundamental frequency; f0beg – the fundamental frequency at the onset of a call; f0end – the fundamental frequency at the end of a call; fpeak – the frequency of maximum amplitude within a call; power-f0 – the relative power of the f0 band compared to the peak harmonic; q25, q50 q75 – the lower, the medium and the upper quartiles, covering respectively 25%, 50% and 75% energy of a call spectrum. The spectrogram was created with Hamming window; 11025 kHz sampling rate; FFT 1024 points; frame 50%; and overlap 96.87%.

This study investigates the structure of the oral and nasal calls of individually identified mother and young Iberian red deer in calving period. We compared the potential of variables of the oral and nasal calls in both mother and young (Figure 1) to encode individual identity. We also tested whether body mass and sex affected the acoustics of calls in calves. We estimate which acoustic variables primarily contribute to vocal identity in the oral and in the nasal calls and across call types. In addition, for hinds, we estimate the between-year stability of individual identity in their oral and nasal contact calls.

## Results

### Comparison of oral and nasal calls

For hinds, a repeated measures ANOVA controlling for individuality revealed no significant differences in the duration of oral and nasal calls, but revealed a significantly shorter dur-to-max in the oral compared to the nasal calls (Table 1). None of the variables associated with f0 differed between oral and nasal calls with the exception of f0beg, which was significantly higher in oral calls. The fpeak, q25, power-f0 and peak-harm were also significantly higher in the oral than in the nasal calls (Table 1).

**Table 1:** **Values (mean ± SD) of oral and nasal call variables and repeated measures ANOVA results for their comparison**

**Call variable**	**Hinds (n = 28)**			**Calves (n = 31)**		
	**Oral calls**	**Nasal calls**	**ANOVA**	**Oral calls**	**Nasal calls**	**ANOVA**
durat (s)	0.759 ± 0.235	0.791 ± 0.253	*F*_1,27_ = 0.69, *p* = 0.414	0.264 ± 0.080	0.230 ± 0.039	*F*_1,30_ = 9.79, *p* = **0.004**
dur-to-max (s)	0.230 ± 0.098	0.308 ± 0.138	*F*_1,27_ = 10.90, *p* = **0.003**	0.311 ± 0.078	0.300 ± 0.133	*F*_1,30_ = 0.16, *p* = 0.696
f0beg (Hz)	137 ± 33	128 ± 29	*F*_1,27_ = 7.40, *p* = **0.011**	773 ± 113	695 ± 122	*F*_1,30_ = 22.97, *p* < **0.001**
f0end (Hz)	89 ± 19	89 ± 16	*F*_1,27_ = 0.15, *p* = 0.700	518 ± 96	481 ± 58	*F*_1,30_ = 4.80, *p* = **0.036**
f0max (Hz)	180 ± 31	173 ± 32	*F*_1,27_ = 3.02, *p* = 0.094	875 ± 99	781 ± 103	*F*_1,30_ = 64.49, *p* < **0.001**
f0min (Hz)	95 ± 18	93 ± 14	*F*_1,27_ = 1.11, *p* = 0.301	554 ± 93	512 ± 76	*F*_1,30_ = 9.06, *p* = **0.005**
f0mean (Hz)	153 ± 25	148 ± 24	*F*_1,27_ = 3.46, *p* = 0.074	778 ± 87	694 ± 79	*F*_1,30_ = 57.39, *p* < **0.001**
∆f0 (Hz)	85 ± 26	80 ± 29	*F*_1,27_ = 1.49, *p* = 0.233	321 ± 77	269 ± 92	*F*_1,30_ = 13.24, *p* = **0.001**
fpeak (Hz)	1418 ± 555	1060 ± 568	*F*_1,27_ = 12.82, *p* = **0.001**	2973 ± 684	2289 ± 948	*F*_1,30_ = 19.39, *p* < **0.001**
q25 (Hz)	952 ± 285	713 ± 250	*F*_1,27_ = 24,50, *p* < **0.001**	1681 ± 506	1163 ± 387	*F*_1,30_ = 28.66, *p* < **0.001**
q50 (Hz)	1731 ± 255	1632 ± 257	*F*_1,27_ = 3.03; *p* = 0.093	2987 ± 384	2488 ± 515	*F*_1,30_ = 30.89; *p* < **0.001**
q75 (Hz)	2471 ± 252	2534 ± 261	*F*_1,27_ = 1.20, *p* = 0.283	3730 ± 238	3564 ± 395	*F*_1,30_ = 7.10, *p* = **0.012**
power-f0 (dB)	13.90 ± 5.76	5.38 ± 4.10	*F*_1,27_ = 55.15, *p* < **0.001**	10.49 ± 5.67	6.90 ± 6.98	*F*_1,30_ = 8.14, *p* = **0.008**
peak-harm	8.2 ± 3.3	6.1 ± 3.1	*F*_1,27_ = 10.66, *p* = **0.003**	3.5 ± 0.9	3.0 ± 1.3	*F*_1,30_ = 4.65, *p* = **0.039**

Designations: durat – call duration; dur-to-max – the duration from call onset to the point of the maximum fundamental frequency; f0beg – the fundamental frequency at the onset of a call; f0end – the fundamental frequency at the end of a call; f0max – the maximum fundamental frequency of a call; f0min – the minimum fundamental frequency of a call; f0mean – the average fundamental frequency of a call; ∆f0 – the depth of frequency modulation, calculated as the difference between f0max and f0 min; fpeak – the frequency of maximum amplitude within a call; q25, q50, q75 – the lower, medium and upper quartiles of a call; power-f0 – the relative power of the f0 band compared to the peak frequency band; peak-harm – the order number of the harmonic with the maximum energy. Significant differences are highlighted in bold.

For calves, a repeated measures ANOVA controlling for individuality revealed significantly higher values in all measured variables in the oral calls compared to the nasal calls with the exception of dur-to-max (Table 1). Unlike hinds, in calves the oral calls were longer and had higher values of f0 variables compared to the nasal calls. Overall, oral and nasal calls differed by structure more strongly in calves than in hinds.

### Individual discrimination with DFA

**Figure 2:**
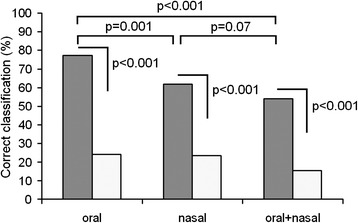
**Individual discrimination of hinds based on oral calls, nasal calls, and the pooled call sample.** Gray bars indicate values of discriminant function analysis (DFA) and white bars indicate random values, calculated with the randomization procedure. Comparisons between observed and random values and between oral calls, nasal calls, and the pooled sample of oral and nasal calls with *χ* ^2^ tests are shown by brackets above. Examples of nasal calls of four individual hinds are given in Additional file 1.

For the same sample of 22 hinds, we estimated the values of correct classification to individual for oral calls, for nasal calls, and for the pooled sample of oral and nasal calls (Figure 2). In all three DFAs, the average value of correct assignment to individual (77.0% for oral calls, 61.8% for nasal calls, 53.9% for the pooled sample of oral and nasal calls) significantly exceeded our random expectation (24.1 ± 2.5%, 23.5 ± 2.6% and 15.5 ± 1.6% respectively, all *p* < 0.001). The average value of correct assignment to individual was higher in oral than in nasal calls (*χ*^2^_1_ = 10.80, *p* = 0.001) and in oral calls than in calls of the pooled sample of oral and nasal calls (*χ*^2^_1_ = 34.57, *p* < 0.001), although it did not differ significantly between nasal calls and the pooled sample of oral and nasal calls (*χ*^2^_1_ = 3.24, *p* = 0.07) (Figure 2).

Because the same variables were included in a DFA for both oral and nasal calls in the same sample of 22 hinds, we could compare percentages of correct assignment for nasal and oral calls to particular individuals with repeated measures ANOVA. The ANOVA revealed significantly higher percentages of correct assignment to individual for oral calls compared to nasal calls (*F*_1,21_ = 10.76; *p* = 0.004) and for oral calls compared to the pooled sample of oral and nasal calls (*F*_1,21_ = 28.63; *p* < 0.001), although it did not reveal statistical differences in classifying accuracy between nasal calls and the pooled sample of oral and nasal calls (*F*_1,21_ = 4.03; *p* = 0.06).

For hind oral calls, the f0max, f0end and f0mean (in order of decreasing importance) were mainly responsible for discrimination of individuals (Table 2). For hind nasal calls, the duration, f0beg and f0mean (in order of decreasing importance) were mainly responsible for discrimination of individuals (Table 2). For hind pooled sample of oral and nasal calls, the f0max, f0mean and duration (in order of decreasing importance) were mainly responsible for discrimination of individuals (Table 2). Therefore, in all three DFAs for hinds, similar sets of cue discriminating variables were found.

**Table 2:** **DFA results for hind calls**

**Call variable**	**Oral calls**		**Nasal calls**		**Oral + Nasal calls**	
	**Wilks’ lambda**	**Variable effect**	**Wilks’ lambda**	**Variable effect**	**Wilks’ lambda**	**Variable effect**
durat	0.678067	*F* = 4.002	**0.639569**	*F* = 4.830	**0.729086**	*F* = 6.883
dur-to-max	0.665226	*F* = 4.242	0.859585	*F* = 1.400	0.828365	*F* = 3.838
f0beg	0.766825	*F* = 2.563	**0.673034**	*F* = 4.164	0.793032	*F* = 4.834
f0end	**0.629795**	*F* = 4.954	0.802595	*F* = 2.108	0.813760	*F* = 4.239
f0max	**0.616740**	*F* = 5.238	0.684320	*F* = 3.954	**0.718309**	*F* = 7.264
f0mean	**0.637517**	*F* = 4.792	**0.679053**	*F* = 4.051	**0.724155**	*F* = 7.056
∆f0	0.650492	*F* = 4.529	0.825325	*F* = 1.814	0.785000	*F* = 5.073
q25	0.772391	*F* = 2.484	0.744996	*F* = 2.934	0.809097	*F* = 4.371
q50	0.829728	*F* = 1.730	0.756659	*F* = 2.757	0.880519	*F* = 2.514
q75	0.661713	*F* = 4.309	0.759728	*F* = 2.711	0.844924	*F* = 3.400
power-f0	0.672220	*F* = 4.110	0.824961	*F* = 1.819	0.897734	*F* = 2.110

The Wilks’ Lambda values and call variable effects are presented for each acoustic variable included in the three independent DFAs for call assignment to individual on the basis of oral calls, nasal calls, and the pooled sample of oral and nasal calls. The smaller the Wilks’ Lambda value, the greater the contribution of the given call variable to the overall discrimination. For each DFA, the three variables that contributed the most to discrimination are highlighted in bold. Designations as in Table 1.

**Figure 3:**
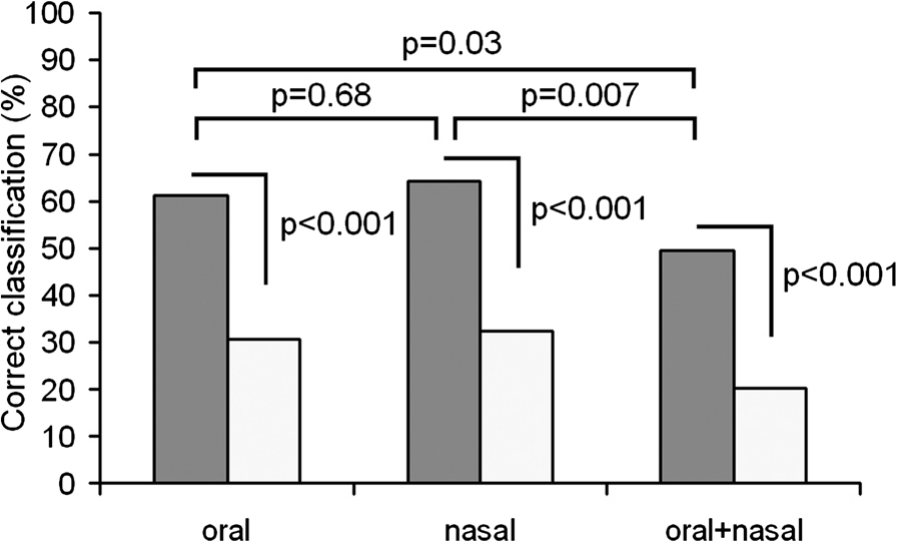
**Individual discrimination of calves based on oral calls, nasal calls, and the pooled call sample.** Gray bars indicate values of discriminant function analysis (DFA) and white bars indicate random values, calculated with randomization procedure. Comparisons between observed and random values and between oral calls, nasal calls, and the pooled sample of oral and nasal calls with *χ* ^2^ tests are shown by brackets above. Examples of oral calls of four individual calves are given in Additional file 2.

For the same sample of 17 calves, we estimated the values of correct classification to individual for oral calls, for nasal calls, and for the pooled sample of oral and nasal calls (Figure 3). In all the three DFAs, the average value of correct assignment to individual (61.1% for oral calls, 64.2% for nasal calls, 49.5% for the pooled sample of oral and nasal calls) exceeded significantly the random value (respectively 30.8 ± 3.5%, 32.2 ± 3.6%, 20.3 ± 2.0%), calculated with randomization procedure (all differences are significant, *p* < 0.001). Unlike hinds, in calves the average value of correct assignment to individual did not differ between oral and nasal calls (*χ*^2^_1_ = 0.17, *p* = 0.68) and was significantly higher either in oral or in nasal calls compared to the pooled sample of oral and nasal calls (*χ*^2^_1_ = 4.83, *p* = 0.03 and *χ*^2^_1_ = 7.34, *p* = 0.007 respectively) (Figure 3).

Because the same variables were included in a DFA for both oral and nasal calls in the same sample of 17 calves, we could compare percentages of correct assignment for nasal and oral calls to particular individuals with repeated measures ANOVA. The ANOVA did not reveal significant differences in percentages of correct assignment to individual between oral and nasal calls (*F*_1,16_ = 0.08; *p* = 0.78). For the pooled sample of oral and nasal calls, the percentages of correct assignment to individual were significantly lower than with unpooled samples of either oral (*F*_1,16_ = 5.09; *p* = 0.04) or nasal calls (*F*_1,16_ = 5.82; *p* = 0.03).

For calf oral calls, the f0beg, duration and power-f0 (in order of decreasing importance) were primarily responsible for discrimination of individuals (Table 3). For calf nasal calls and for the pooled sample of oral and nasal calls, the f0beg, duration and q75 (in order of decreasing importance) were primarily responsible for discrimination of individuals (Table 3). Thus, in all three DFAs for calves, the sets of variables primarily responsible for discriminating individuals were similar.

**Table 3:** **DFA results for calf calls**

**Call variable**	**Oral calls**		**Nasal calls**		**Oral + Nasal calls**	
	**Wilks’ lambda**	**Variable effect**	**Wilks’ lambda**	**Variable effect**	**Wilks’ lambda**	**Variable effect**
durat	**0.632018**	*F* = 4.440	**0.668077**	*F* = 3.323	**0.674678**	*F* = 7.715
dur-to-max	0.839782	*F* = 1.455	0.776154	*F* = 1.929	0.886460	*F* = 2.049
f0beg	**0.498984**	*F* = 7.656	**0.447194**	*F* = 8.267	**0.545710**	*F* = 13.320
f0end	0.830615	*F* = 1.555	0.858480	*F* = 1.102	0.904361	*F* = 1.692
f0max	0.783864	*F* = 2.102	0.772026	*F* = 1.975	0.814221	*F* = 3.651
f0mean	0.779072	*F* = 2.162	0.820857	*F* = 1.459	0.816577	*F* = 3.594
∆f0	0.934081	*F* = 0.538	0.854374	*F* = 1.140	0.953091	*F* = 0.787
q25	0.832533	*F* = 1.534	0.784757	*F* = 1.834	0.890603	*F* = 1.965
q50	0.886230	*F* = 0.979	0.837572	*F* = 1.297	0.929386	*F* = 1.216
q75	0.754355	*F* = 2.483	**0.668245**	*F* = 3.320	**0.811584**	*F* = 3.715
power-f0	**0.643181**	*F* = 4.230	0.764927	*F* = 2.055	0.854513	*F* = 2.724

The Wilks’ Lambda values and variable effects are presented for each acoustic variable included in the three independent DFAs for call assignment to individual in calves on the basis of oral calls, nasal calls, and the pooled sample of oral and nasal calls. The smaller the Wilks’ Lambda value, the greater the contribution of the given call variable to the overall discrimination. For each DFA, the three variables that contributed the most to discrimination are highlighted in bold. Designations as in Table 1.

Body mass of 22 hinds whose calls were included in DFA was mean ± SD = 104.8 ± 12.2 kg and ranged from 85.3 to 121.5 kg, and body mass of 17 calves whose calls were included in DFA was 16.0 ± 4.4 kg and ranged from 10.0 to 25.8 kg. To approximate probable effects of body mass on the acoustic variables, for hinds we calculated correlations between log_3_ body mass and five variables (durat, f0beg, f0end, f0max, f0mean, see Table 2) primarily contributing to discrimination of individuals by calls. We did not find effects of body mass on variables of either oral calls (durat: *r* = 0.12, *p* =0.61; f0beg: *r* = −0.20, *p* =0.38; f0end: *r* = −0.27, *p* =0.24; f0max: *r* = −0.13, *p* =0.55; f0mean: *r* = −0.23, *p* = 0.31) or nasal calls (durat: *r* = 0.08, *p* = 0.74; f0beg: *r* = −0.26, *p* = 0.24; f0end: *r* = −0.20, *p* = 0.38; f0max: *r* = −0.14, *p* = 0.55; f0mean: *r* = −0.21, *p* = 0.34).

For calves, we estimated the effects of sex, body mass and the conjoint effect of sex*body mass on four acoustic variables (durat, f0beg, q75, power-f0, see Table 3) primarily contributing to discrimination of individuals by calls. ANCOVA with sex as a fixed categorical factor and body mass as a continuous factor revealed significant effects of body mass on the duration, f0beg and q75 of the oral calls and on the duration of the nasal calls (Table 4). For all these acoustic variables the values decrease with increase of body mass. Only for the nasal calls ANCOVA revealed significant effects of sex and sex*body mass on the duration and q75 (Table 4), which values were lower in male than in female calves (duration: males 0.227 ± 0.019 s, females 0.232 ± 0.031 s; q75: males 3588 ± 258 Hz, females 3642 ± 243 Hz). At the same time, body mass did not differ significantly (one-way ANOVA, *F*_1,16_ = 1.15, *p* = 0.30) between male and female calves (males 17.0 ± 4.9 kg, females 14.7 ± 3.7 kg).

**Table 4:** **ANCOVA results for calf call variables**

**Call variable**	**Oral calls**			**Nasal calls**		
	**Sex effect**	**Body mass effect**	**Sex & body mass effect**	**Sex effect**	**Body mass effect**	**Sex & body mass effect**
durat	*F*_1,13_ = 1.66, *p* = 0.220	*F*_1,13_ = 8.06, ***p*** **= 0.014**	*F*_1,13_ = 1.37, *p* = 0.262	*F*_1,13_ = 4.88, ***p*** **= 0.046**	*F*_1,13_ = 29.10, ***p*** **< 0.001**	*F*_1,13_ = 5.16, ***p*** **= 0.041**
f0beg	*F*_1,13_ = 0.23, *p* = 0.642	*F*_1,13_ = 8.46, ***p*** **= 0.012**	*F*_1,13_ = 0.20, *p* = 0.660	*F*_1,13_ = 0.14, *p* = 0.716	*F*_1,13_ = 4.05, *p* = 0.065	*F*_1,13_ = 0.10, *p* = 0.755
q75	*F*_1,13_ = 3.88, *p* = 0.070	*F*_1,13_ = 5.70, ***p*** **= 0.033**	*F*_1,13_ = 3.29, *p* = 0.093	*F*_1,13_ = 7.83, ***p*** **= 0.015**	*F*_1,13_ = 0.50, *p* = 0.491	*F*_1,13_ = 8.15, ***p*** **= 0.014**
power-f0	*F*_1,13_ = 0.81, *p* = 0.385	*F*_1,13_ = 3.40, *p* = 0.088	*F*_1,13_ = 1.45, *p* = 0.250	*F*_1,13_ = 0.92, *p* = 0.354	*F*_1,13_ = 2.65, *p* = 0.127	*F*_1,13_ = 1.33, *p* = 0.269

Sex is taken as a fixed categorical factor and body mass as a continuous factor**.** Designations as in Table 1. Effects considered significant are highlighted in bold.

### Between-year stability of hind calls

**Figure 4:**
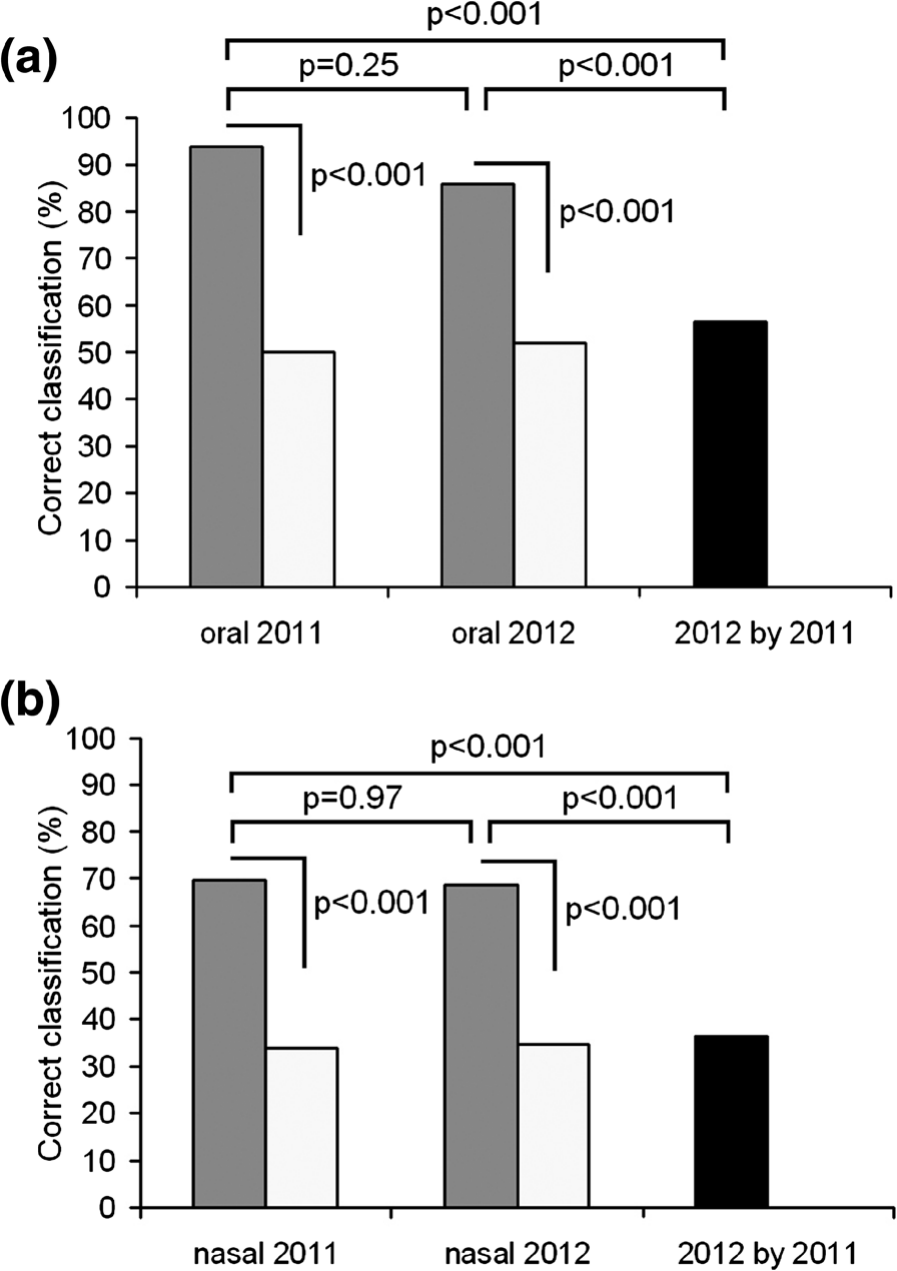
**Discrimination of individual hinds by oral (a) and nasal (b) calls in two consequent years.** Gray bars indicate values of discriminant function analysis (DFA) and white bars indicate random values, calculated with a randomization procedure. Comparisons between observed and random values and between 2011 and 2012 calls with *χ* ^2^ tests are shown by brackets above. The black bar indicates the classification value of 2012 calls with discriminant functions created for 2011 calls.

For hinds that provided sufficient number of calls in both study years (2011 and 2012) we compared the stability of vocal individual identity between years (Figure 4). Within years, individuality was very high in either oral or nasal calls. Among oral calls, the average values of correct classification to individual (93.8% in 2011 and 86.0% in 2012) significantly exceeded the random expectation (49.9 ± 5.5% in 2011 and 52.0 ± 6.2% in 2012, *p* < 0.001 in both cases) and did not differ between years (*χ*^2^_1_ = 1.33, *p* = 0.25). Among nasal calls, the average values of correct classification (69.6% in 2011 and 68.6% in 2012) significantly exceeded the random expectation (33.6 ± 3.6% in 2011 and 34.7 ± 3.8% in 2012, *p* < 0.001 in both cases) and did not differ between years (*χ*^2^_1_ = 0, *p* = 0.97).

However, cross-validation of calls recorded in 2012 using discriminant functions created for calls recorded in 2011 revealed a strong decrease in the correct classification of individuals with either oral or nasal calls (Figure 4). For the oral calls, the average value of correct classification dropped to the level expected by chance alone (56.1%), and became significantly lower compared to call samples from either 2011 (*χ*^2^_1_ = 21.79, *p* < 0.001) or from 2012 (*χ*^2^_1_ = 10.92, *p* = 0.001). Similarly, for the nasal calls, the value of correct classification dropped to the level expected by chance alone (36.4%) and became significantly lower compared to call samples from either 2011 (*χ*^2^_1_ = 25.96, *p* < 0.001) or from 2012 (*χ*^2^_1_ = 23.93, *p* < 0.001). Thus, in hinds, individual identity of oral and nasal calls was unstable between years.

## Discussion

### The acoustics of oral and nasal calls

Our study revealed that among calves, the fundamental frequency was higher in oral than in nasal calls. The same relationship was previously found in calls of juvenile goitred gazelles [31], in rumbles of adolescent African elephants *Loxodonta africana* [50], in calls of adult female domestic sheep [35], and in calls of newborn and adult female saiga antelopes [36]. The higher fundamental frequency of oral than nasal calls may represent a common rule, in that oral calls are made at higher arousal. The anatomical and productional basis of this difference in fundamental frequency between oral and nasal calls in mammals has been discussed in detail in previous studies [31,36]. Nevertheless, in Iberian red deer hinds in this study, the fundamental frequency did not differ between oral and nasal calls. These results contradict those obtained for other ungulates [31,35,36] and confirm our previous data for unmarked Iberian red deer hinds during the rut [37].

The values of fundamental frequency variables of Iberian red deer calf oral calls in our study (f0max = 875 Hz, Table 1) were similar to those reported for oral distress calls of neonate Middle-European red deer *C.e. hippelaphus* (f0max = 844 Hz [23]), and were slightly higher in frequency than distress calls of 2–4 day-old Middle-European red deer calves (f0max = 737 Hz [28]) and contacts calls of 4 month-old Corsican red deer calves *C.e. corsicanus* (f0max = 710 Hz [51]). At the same time, power quartiles of calf oral calls in our study (q25 = 952 Hz, q50 = 1731 Hz, q75 = 2471 Hz, Table 1) were substantially lower than on [23] (q25 = 2385 Hz, q50 = 4669 Hz, q75 = 7398 Hz). These differences could be attributable to degree of emotional arousal of a caller, as we collected contact calls from calves that were separated from their mothers, whereas Teichroeb et al. [23] collected distress calls made during captures, ear tagging and human handling. The energy shifts towards higher frequencies (and respective increase of power quartile values) represent a common rule of acoustics changes attending increased emotional arousal of mammalian callers [52-54].

Compared to calls of 3–4 month-old calves recorded on the same farm during the rut [37], calls during the calving period were shorter and higher-pitched, which is a product of decreasing of fundamental frequency with age. Hind calls were higher in fundamental frequency during the rut relative to the calving period, which may result from the lower disturbance of animals in the current study because of the longer period of data collection. In both the rut [37] and calving period (this study) we found a higher fundamental frequency in oral than in nasal calf calls and did not observe significant differences between oral and nasal calls of hinds. Our study also confirmed the findings of previous studies of Iberian red deer, that revealed that the fundamental frequency of hind contact calls is lower than that in rutting roars of stags [37,55].

### Individuality of oral and nasal calls

In hinds, individual identity was more pronounced in oral than in nasal calls. The same relationship was previously reported for calls of juvenile goitred gazelles [31]. One possible explanation for the higher potential of oral calls to allow deer to discriminate different individuals is that such calls are produced in situations of higher tension than nasal calls [31,35]. Among Iberian red deer calves in our study, however, the expression of individual identity detected with DFA, did not differ between oral and nasal calls. No comparative data is available to explain this discrepancy.

In hinds and in calves, classification accuracy of callers was two to three times greater than the random expected value. This was observed even for the pooled sample of oral and nasal calls, probably because the same acoustic variables were responsible for individuality of either oral or nasal calls. This should allow mothers and their young to remember only a single set of acoustic variables for mutual vocal recognition instead of remembering two different sets for the oral and for the nasal calls. At the same time, variables mostly contributing to discrimination differed between mother and young. In mothers, discriminating power of oral and nasal calls was mainly based on variables related to fundamental frequency, whereas in calves, it included f0beg along to the duration and power-f0, that is, was based on variables of all modalities, temporal, frequency and power. To reveal the communicative functions of nasal and oral calls, further playback study would be necessary. Consistent with our demonstration of individually-distinct contact calls among hinds and calves, orally produced rutting roars of red deer stags also have a potential to encode individual identity. In two studies, the degree of correct classification of individual stags by their rutting roars was about 70% for a sample of 5–6 stags, in spite of different sets of measured acoustic variables [47,48].

Estimating vocal individuality was complicated by differences in age ranges among calves during the data collection period. Call parameters, in particular the fundamental frequency, depend on the caller’s body size among ungulates [34,56], although in adult red deer this dependence is lacking [37,57]. The fundamental frequency parameters contributed substantially to classifying individuals with DFA in both calves and hinds. Therefore, to address this issue, we repeatedly recorded individuals across the period of data collection. Nevertheless, we found significant effects of body mass on the start fundamental frequency and duration in the oral calls and on duration in the nasal calls of calves. Thus, we could not totally exclude the effects of the body mass on individual distinctiveness of calf contact calls in this study. Nevertheless, the vocal individuality of calves was comparable with that of hinds (in which effects of body mass on call variables were not found) and with values in other ungulates, e.g. 20 goitred gazelle calves, with correctly classified to individual 75% oral calls and 65% nasal calls [31].

### Between-year stability of hind calls

Individual identity of hind oral and nasal calls was unstable between years. Thus, if the ability to discriminate among individuals based on calls alone is to persist across years, cues to individual identity in hind oral and nasal calls must develop anew each year. Degradation of vocal individuality with time has been demonstrated for common roars of red deer stags [46] and for groans of fallow deer bucks [45]. These results support the hypothesis that vocal individuality does not result exclusively from individual vocal anatomy and that there is some vocal tuning and vocal flexibility in vocalizations of ungulates [32,58,59].

## Materials and methods

### Study site, subjects and dates of recordings

Calls of hinds and calves were recorded in June 2011 and 2012 at the farm of the University of Castilla-La Mancha (Albacete, Spain, 38°57’10”N, 1°47’00”W, 690 m a.s.l.). The population originated in 1994 from 15 male and 50 female pure Iberian deer from a nearby Las Dehesas public game reserve in Alpera (Albacete) and from Cabaneros National Park (Toledo). The animals used in this study were born and kept in four 10,000 m^2^ enclosures on an irrigated pasture. They were fed *ad libitum* with a diet of barley straw and meal from barley, alfalfa, oats and sugar beets [60].

All mothers and calves were captive-born and kept together in permanent groups (4 groups in 2011 and 3 groups in 2012) separately from adult stags and yearlings. The groups ranged in size from 6 hinds and 2 calves to 30 hinds and 24 calves (mean ± SD = 17.4 ± 7.6 hinds and 15.0 ± 7.7 calves per group). The entire population of animals from which we collected calls counted 61 hinds and 52 calves in 2011 and 61 hinds and 53 calves in 2012 (45 hinds were the same in both years). The age of mothers was 2–19 years (mean ± SD = 10.0 ± 4.6 years). The calves were born from 6 May to 23 June in 2011 and from 13 May to 14 June in 2012. During data collection, the age of calves varied of 1–52 days. All study calves were singletons, excluding two sibling pairs, one in 2011 and one in 2012.

All hinds and calves were individually labeled with Allflex (Palmerston North, New Zealand) plastic ear tags and all hinds were additionally marked with the Allflex color collars with numbers. Three times a day, the farm staff inspected each enclosure with hinds and calves for newborns and labeled them individually with an ear tag, which allowed us to establish a mother for each calf.

### Call and body mass collection

For acoustic recordings of hind and calf (48 kHz, 16 bit), we used a solid state recorder Marantz PMD-660 (D&M Professional, Kanagawa, Japan) with a Sennheiser K6-ME66 cardioid electret condenser microphone (Sennheiser electronic, Wedemark, Germany). The distance from the hand-held microphone to the animals was 5–35 m, the level of recording was adjusted during the recordings accordingly to the intensity of the produced calls.

We recorded calls daily, from 6:00–7:00 to 12:00–13:00, often with synchronous video, using a digital camcorder Panasonic HDC-HS100 (Panasonic Corp., Kadoma, Japan). During recordings, individual identities of callers producing calls through the mouth and through the nose were labeled by voice. Recordings have been conducted both inside and outside the outdoor enclosures during different contexts: everyday routine activity, when mothers searched for their offspring which were hidden in the enclosures; at translocations to small paddocks, from where the animals were taken for weightings; during temporal separations of hinds and calves after the weighing and at short separations by presence of researchers in enclosure on the way between a mother and her calf. In total, in 2011 and 2012, we collected 30 hours of audio recordings (16 hours in 2011 and 14 hours in 2012) from 28 individual hinds (9 hinds were recorded in both 2011 and 2012) and from 31 individual calves. All animals were weighted one time with Mettler-Toledo ID1 scales (Mettler-Toledo S.A.E., Barcelona, Spain) as the part of routine farm management [61] during the periods of acoustic recordings. The age of calves at weightings ranged from 1 to 40 days.

### Acoustic analyses

For acoustic analyses, we took only calls of good quality with high signal-to-noise ratios sufficient for analysis of all acoustic variables, measured in this study that were not disrupted by wind or the calls of other animals or overloaded during the recordings. We analysed only individually identified calls of known call type (nasal or oral). Calls were classified to nasal and oral call types based on voice comments of researchers made during recording, by video clips, made synchronously with the recordings, by the obvious nasal quality of sound within a recording (Additional files 1 and 2) and by the difference in call energy distribution, shifted towards higher frequencies in oral calls due to the shortening the vocal tract at opening of the mouth. These methods of classification to nasal and oral call types were previously applied for the Iberian red deer [37], for goitred gazelles [31,32] and for saiga antelopes [36]. Two researchers (OS and IV) independently classified all calls, and we took for analysis only calls where both researchers were concordant in their judgments concerning their type. To reduce pseudoreplication, we took calls from different recording sessions per animal and from different parts within session, because calls from the same sequence are commonly more similar in their acoustic structure than calls from different sequences [62]. The mean ± SD number of sessions per animal was 5.3 ± 4.4, and we took up to 15 high-quality calls per individual per call type (nasal and oral) from 28 mothers and 31 young (16 males, 15 females). We analysed 801 calls of mothers (354 oral and 447 nasal) and 469 calls of calves (281 oral and 188 nasal), for 1270 calls in total.

Acoustic analyses were conducted in the same way for hinds and calves and for the oral and nasal calls. For each nasal and oral call, we measured the same set of 14 acoustic variables: 2 temporal, 6 variables of fundamental frequency (f0) and 6 power variables, as they proved their use for estimating vocal individual identity in red deer [28,47,48]. Before analysis, the calls were downsampled to 11025 Hz and high-pass filtered at 50 Hz, to increase frequency resolution and to reduce the low-frequency background noise. We measured the duration of each call and the duration from call onset to the point of maximum f0 (dur-to-max) manually on the screen with the reticule cursor in the spectrogram window (Hamming window, FFT 1024 points, frame 50% and overlap 96.87%) by using Avisoft SASLab Pro software (Avisoft Bioacoustics, Berlin, Germany). Then we performed manual measurements on the screen with the standard marker cursor of the start (f0beg), maximum (f0max) and end (f0end) fundamental frequencies of each call (Figure 1). Measurements were exported automatically to Microsoft Excel (Microsoft Corp., Redmond, WA, USA).

In a 50 ms call fragment symmetrical about f0 maximum, we created the power spectrum, from which we automatically measured fpeak, representing the value of the frequency of maximum amplitude and the q25, q50 and q75, representing the lower, medium and upper quartiles, covering 25%, 50% and 75% of the energy of the call spectrum respectively (Figure 1). On the same spectrum, we estimated (in dB) the power-f0, representing the relative power of the f0 band compared to the peak harmonic, on the screen using two harmonic cursors (Figure 1). The power-f0 was equal to 0 when the f0 band coincided with the fpeak band. In addition, we recorded the peak-harm, representing the order number of the harmonic with the maximum energy.

In addition, we measured the f0 variables following Reby and McComb [63] by using the Praat DSP package [64]. The f0 contour was extracted by using a cross-correlation algorithm (to Pitch (cc) command in Praat). The time steps in the analysis were 0.01 s for hinds and 0.005 s for calves; the lower and upper limits of the f0 range were 50–400 Hz for hinds and 100–1200 Hz for calves. A preliminary visual analysis of the spectrograms in Avisoft showed that the lower limit was lower than the minimum f0 for calls of either hinds or calves. Spurious values and octave jumps in the f0 contour were corrected manually on the basis of the spectrograms. Values of f0min, f0max, the depth of frequency modulation f0 (∆f0 = f0max - f0min) and average f0 of a call (f0mean) were taken automatically by using by using the Pitch info command in the Pitch edit window.

Two different methods of measuring f0max (one using Avisoft and another using Praat) applied to the same calls, resulted in very similar values. Coefficients of correlation, calculated separately for the oral and for the nasal calls of hinds and calves, ranged between 0.994 and 0.996 (0.988 < R^2^ < 0.992). Thus, for subsequent acoustic analyses we could select between these methods and we used the f0 values measured with Praat.

We did not measure formants, as they can only be measured either in low-frequency calls with closely spaced harmonics or in noisy calls (e.g. stag harsh roars), where the sound energy is dispersed over the call spectrum [26,37,55]. In most calls of hinds and in all calls of calves, formant frequencies could not be measured because they fell either between the harmonics and were thus invisible, or they were indistinguishable from the harmonics because they coincided with them in frequency.

### Call samples and statistical analyses

Statistical analyses were conducted using STATISTICA v. 6.0 (StatSoft, Tulsa, OK, USA) and R v.3.0.1 [65]. Means are given as mean ± SD, all tests were two-tailed, and differences were considered significant whenever *p* < 0.05. Distributions of most measured parameter values (excepting fpeak and peak-harm) did not depart from normality and distributions of all mean parameter values did not depart from normality (Kolmogorov-Smirnov test, *p* > 0.05). As parametric ANOVA and discriminant function analysis (DFA) are relatively robust to departures from normality [66], this was not an obstacle to the application of these tests.

We used a repeated measures ANOVA controlled for individuality, to compare the parameter values between oral and nasal calls. We used 1–15 (9.3 ± 5.2) calls per animal per call type, from 28 hinds (300 oral and 325 nasal calls) and from 31 calves (16 males, 15 females; 281 oral and 188 nasal calls). From hinds which provided calls in both years, we took calls recorded only in one of the years (2011 or 2012). For oral and nasal calls of these 28 hinds and 31 calves, we calculated average values of acoustic variables.

We used DFA to calculate the probability of the assignment of calls to the correct individual for three call samples in both hinds and calves (of nasal calls, oral calls and the pooled sample of oral and nasal calls). We took 5–10 (9.0 ± 1.5) calls per animal per call type from 22 hinds (209 oral and 212 nasal calls in total) and from 17 calves (10 males, 7 females; 149 oral and 134 nasal calls in total). DFA requires balanced sample sizes per group, and thus we excluded from the analyses all the animals with less than 5 oral/nasal calls. We took all the calls from all the animals with from 5 to 10 oral/nasal calls and randomly selected 10 calls per type from animals with more than 10 measured calls of each type. We included 11 of the 14 measured call variables in the DFA, excluding fpeak and peak-harm (for not meeting the criterion of normality), and f0min (because it was used for calculating another variable).

Then we investigated the stability of acoustic individuality of hind oral and nasal calls between years for hinds that provided calls in both years. We classified hind calls from 2012 year with DFA functions derived from 2011, considering the value of the correct cross-validation as a measure of the retention of individuality over time [39,43,44,67]. We used 7–15 (12.2 ± 3.4) oral calls per animal per year from 5 hinds, which provided a sufficient number of oral calls in both years (in total 65 oral calls in 2011 and 57 oral calls in 2012). Also, we used 10–15 (13.7 ± 1.8) nasal calls per animal from 9 hinds which provided sufficient numbers of nasal calls in both years (in total 125 nasal calls in 2011 and 121 nasal calls in 2012).

We used Wilks’ Lambda values to estimate how strongly acoustic variables of calls contribute to the discrimination of individuals. With a 2×2 Yates’ chi-squared test, we compared the values of correct assignment of nasal and oral calls to the correct individual. We used a repeated measures ANOVA to compare the percentages of correct assignment of oral and nasal calls to particular individuals. To validate our DFA results, we calculated the random values of correct assignment of calls to individual by applying randomization procedure with macros, created in R. The random values were averaged from DFAs performed on 1000 randomized permutations on the data sets as described by [68]. For example, to calculate the random value of classifying oral calls to individual hinds, each permutation procedure included the random permutation of 209 calls among 22 randomization groups, respectively to 22 individual hinds which were examined, and followed by DFA standard procedure built-in in STATISTICA. All other permutation procedures were made similarly. Using a distribution obtained by the permutations, we noted whether the observed value exceeded 95%, 99% or 99.9% of the values within the distribution [68]. If the observed value exceeded 95%, 99% or 99.9% of values within this distribution, we established that the observed value did differ significantly from the random one with a probability *p* < 0.05, *p* < 0.01 or *p* < 0.001 respectively [44,68,69].

Because body mass should theoretically be proportional to the cube of a linear dimension like body size, for hinds we used log_3_ body mass to calculate Pearson’s correlation between body mass and acoustic variables of oral and nasal calls. For calves we used ANCOVA (with sex as a fixed categorical factor and body mass as continuous factor) for estimating effects of sex, body mass and the conjoint effect of sex*body mass on acoustic variables of oral and nasal calls. We used one-way ANOVA to compare body mass values between male and female calves.

## Conclusions

### Conclusion 1

Differences in fundamental frequency between oral and nasal calls are age-specific in Iberian red deer. This finding is new for mammals and probably may be found in other Cervidae species. The higher fundamental frequency of oral than nasal calls of red deer young is consistent with available data on three Bovidae and one Elephantidae species [31,35,36,50], whereas the oral and nasal calls of hinds do not fit to this proposal common rule. The role of factors responsible for this discrepancy, e.g. differences in emotional arousal between mother and offspring red deer during brief separations, should be tested by further studies with controlled experimental conditions [e.g. 24,35].

### Conclusion 2

Differences in power to discriminate individuals between oral and nasal calls are age-specific in Iberian red deer. Whereas in both hinds and calves, both oral and nasal call types allow accurate discrimination of individuals with DFA, the more pronounced individuality of oral calls compared to nasal calls was found only in hinds. Whereas the higher individuality of oral calls was found also in one Bovidae species [31], the same individuality of oral and nasal calls has no analogies in mammals.

### Conclusion 3

Hind calls were unstable between seasons. These results indicate that the vocal individuality is not supported automatically even in mature animals which stopped their growth. This may point also on some costs of supporting vocal individuality over long time. Thus, vocal individuality is not permanently supported in hinds, but re-stores on the basis of new combinations of acoustic traits [32].

## Additional files

Additional file 1:
**Examples of nasal calls of four individual hinds, two calls per individual.**
Additional file 2:
**Examples of oral calls of four individual calves, two calls per individual.**

## Abbreviations

DFA: Discriminant function analysis
f0: The fundamental frequency
durat: Call duration
dur-to-max: The duration from call onset to the point of the maximum fundamental frequency
f0beg: The fundamental frequency at the onset of a call
f0end: The fundamental frequency at the end of a call
f0max: The maximum fundamental frequency of a call
f0min: The minimum fundamental frequency of a call
f0mean: The average fundamental frequency of a call
∆f0: The depth of frequency modulation, calculated as the difference between f0max and f0 min
fpeak: The frequency of maximum amplitude within a call
q25: The lower quartile of a call, covering 25% of call energy
q50: The medium quartile of a call covering 50% of call energy
q75: The upper quartiles of a call covering 75% of call energy
power-f0: The relative power of the f0 band compared to the peak frequency band
peak-harm: The order number of the harmonic with the maximum energy
